# Return of Professor James P. White

**Published:** 1866-09

**Authors:** 


RETURN OF PROFESSOR JAMES P. WHITE.—We are invited to announce that Prof. White is on his return voyage, and will be in Buffalo in time to deliver his usual course of lectures upon Obstetrics and the Diseases of Women and Children in the Buffalo Medical College the approaching winter.

We see it stated that since the brief campaign in which Prussia has been so triumphant, Austria has joined the Association for the relief of suffering on the battle-field. It will be remembered that before the war she was the only Christian nation of Europe which held back from joining this truly humane organization.

